# Interprofessional collaboration in nursing homes (interprof): a grounded theory study of general practitioner experiences and strategies to perform nursing home visits

**DOI:** 10.1186/s12875-016-0522-z

**Published:** 2016-08-30

**Authors:** Nina Fleischmann, Britta Tetzlaff, Jochen Werle, Christina Geister, Martin Scherer, Siegfried Weyerer, Eva Hummers-Pradier, Christiane A. Mueller

**Affiliations:** 1 Department of General Practice, University Medical Center Göttingen, Humboldtallee 38, 37073 Göttingen, Germany; 2 Department of Primary Medical Care, University Medical Center Hamburg-Eppendorf, Martinistraße 52, 20246 Hamburg, Germany; 3 University of Applied Sciences and Arts, Faculty V – Health, Religious Education, Social Affairs, Blumhardtstraße 2, 30625 Hannover, Germany; 4 Psychiatric Epidemiology and Demographic Change, Central Institute of Mental Health, Medical Faculty Mannheim/Heidelberg University, J5, 68159 Mannheim, Germany

**Keywords:** Grounded theory, General practitioners, Interdisciplinary communication, Nursing homes, Primary health care, Physician-nurse relations, Qualitative research, Residential facilities

## Abstract

**Background:**

Interprofessionalism, considered as collaboration between medical professionals, has gained prominence over recent decades and evidence for its impact has grown. The steadily increasing number of residents in nursing homes will challenge medical care and the interaction across professions, especially nurses and general practitioners (GPs). The nursing home visit, a key element of medical care, has been underrepresented in research. This study explores GP perspectives on interprofessional collaboration with a focus on their visits to nursing homes in order to understand their experiences and expectations. This research represents an aspect of the interprof study, which explores medical care needs as well as the perceived collaboration and communication by nursing home residents, their families, GPs and nurses. This paper focusses on GPs’ views, investigating in particular their visits to nursing homes in order to understand their experiences.

**Methods:**

Open guideline-interviews covering interprofessional collaboration and the visit process were conducted with 30 GPs in three study centers and analyzed with grounded theory methodology. GPs were recruited via postal request and existing networks of the research partners.

**Results:**

Four different types of nursing home visits were found: visits on demand, periodical visits, nursing home rounds and ad-hoc-decision based visits. We identified the core category “productive performance” of home visits in nursing homes which stands for the balance of GPs´ individual efforts and rewards. GPs used different strategies to perform a productive home visit: preparing strategies, on-site strategies and investing strategies.

**Conclusion:**

We compiled a theory of GPs home visits in nursing homes in Germany. The findings will be useful for research, and scientific and management purposes to generate a deeper understanding of GP perspectives and thereby improve interprofessional collaboration to ensure a high quality of care.

## Background

Interprofessionalism, considered as collaboration among the health professions, has gained prominence over recent decades [1, 2] and evidence for its impact has grown [3–5]. Interprofessionalism is built on common perceptions, understanding and the effectiveness of working relationships [6]. The increasing number of residents in nursing homes will challenge medical care and the interaction across the professions, especially nurses and general practitioners (GPs). In Germany, currently 29 % of people needing long-term care live in more than 13,000 nursing homes [7]. Most of them suffer from chronic diseases and multimorbidity [8], which results in a complex care requirement and reliance on GPs, who provide coverage for this patient group in Germany and are usually self-employed in either single-handed or in small group practices with 2–4 physicians. Although nursing home residents are free to choose their GP by law, in practice the selection is often made by nurses [9–11]. Nursing homes are allowed to make firm arrangements with GPs [12], but only a quarter of these, generally the smaller nursing homes, achieve this. Even less frequently is a medical practice located within a nursing home [13]. International studies have found various forms of collaboration between nursing homes and GPs, ranging from weekly visits to difficulties even scheduling GPs to visit the nursing home [14]. In Germany, on average, 23 physicians visit a single nursing home. Together with the nurses´ working shift pattern this results in a wide variety of collaboration patterns [12]. A GP typically spends 1.7 h/week in a nursing home, caring for up to 20 residents in this period [13]. Nursing home staff usually consist of specialized geriatric nurses with three years of professional education and training, and nursing aids as well as temporary staff with or with-out vocational training [15].

Previous studies in Germany have mainly focused on either the frequency of home visits [10] or the health status of the residents using quantitative surveys or secondary data analysis [16, 17]. Only a few authors have analyzed the structure of home visits and the interprofessional collaboration between GPs and nurses, where is has been found that agreements between the professional groups, reliable contact persons and periodic visits at fixed times, as well as limiting the number of GPs caring for one nursing home, were essential factors for successful collaboration [18]. From the GP perspective, standardized communication, training, case conferences or more nursing staff presented opportunities for improvement in communication [19]. Further issues for GPs included complaints regarding the working conditions e.g. insufficient remuneration as stipulated by the German medical fee schedule and the degree of collaboration with qualified nurses [20].

This research was part of the interprof study, which explores medical care needs as well as the perceived collaboration and communication by nursing home residents, their families, GPs and nurses. The interprof study aims to uncover limitations and opportunities in interprofessional collaboration thereby permitting development of a model for improvement [21]. This particular project explores GP perspectives on interprofessional collaboration with a focus on their visits to nursing homes in order to understand their experiences and expectations. We describe the core category, its context and influencing conditions as well as the related strategies and consequences [22].

## Methods

### Research design

In this Grounded Theory Study we used open guideline interviews [23] to explore the experiences and expectations of GPs concerning interprofessional collaboration during home visits to nursing homes (Table 1).

**Table 1:** Interview guideline

Narrative of a typical home visit in nursing homes	You have been working here in a nursing home for some time. Today we are interested in your experience of how a nursing home visit is usually carried out. Tell us about typical situations as well as positive and negative experiences during the visits? Can you describe exemplary situations?
Description of the last GP visit	Could you please recall your last home visit to a nursing home resident? How did this particular visit go? Please describe the visit in detail.
Experience with areas of responsibility and distribution of tasks	We are interested in the distribution of tasks in a nursing home. What are your tasks and responsibilities, and what are the tasks and responsibilities of the nursing home staff?
Ideas and vision about the ideal care in a nursing home	Give your imagination free reign. How do you imagine ideal medical care in a nursing home would be provided? What would you like to see; also in the case if your parents were residents there? What do you think the nurses would expect? What process might be optimal for the nurses? And what processes would be best for the residents and what would they likely prefer?

### Participants

Purposive sampling was used to recruit GPs (*n* = 30) in three study centers: Goettingen, Hamburg and Mannheim as well as their surrounding areas. The diversity of sites allowed participant recruitment from both urban and rural settings. To gain a broad insight, we also recruited GPs from a range of demography’s (see Table 2). Participants provide care to 4 to 250 residents in one to ten nursing homes. The hours per week spent attending a nursing home varied from 1 to 30, two GPs answered “every day” and “as required”.

**Table 2:** Demographic details of participating GPs

Characteristics	Number included
Gender	
male	21
female	9
Age	
36–40	2
41–50	8
51–60	12
61–71	8
Years of work experience as a physician	
11–15	4
16–20	5
21–25	6
26–30	5
31–38	10

GPs were recruited via postal requests and using existing contacts of the research institutes and at conferences, with the goal of exploring and extending the theoretical construct of the emergent theory and to capture diverse experiences. Inclusion criteria were sufficient German language skills and working as a GP in the community. Respondents were provided with detailed information and an interview appointment scheduled. Given that participation was voluntary, we did not collect information regarding non-participation.

### Data collection

Open guideline interviews were conducted by four female researchers (NF, BT, CAM and a doctoral student, not an author) and three male researchers (JW and two others, not authors) with different professional backgrounds (nursing science, occupational therapy, medicine/public health, gerontology, sports science, sociology) and aged 28 to 44 years. All interviewers were trained repeatedly by experienced qualitative researchers (including CG) in interview techniques and theoretical background. Face-to-face interviews were carried out in German language, between September 2012 and December 2013 mainly in GPs´ practices and occasionally in the research departments of the respective study centers. The researchers’ professions or their assumptions were not shared with the interviewees. Field notes were written after each interview and memos during the analysis process. Interviews were digitally audio-recorded, transcribed verbatim and checked against the original recordings. For publication purposes interview quotes were translated by an English native speaker and reviewed by the authors. MAXQDA 10 (Qualitative Data Analysis Software) facilitated data analysis. Information on the rigor of the procedure can be found in our study protocol [21].

### Data analysis

**Fig. 1:**
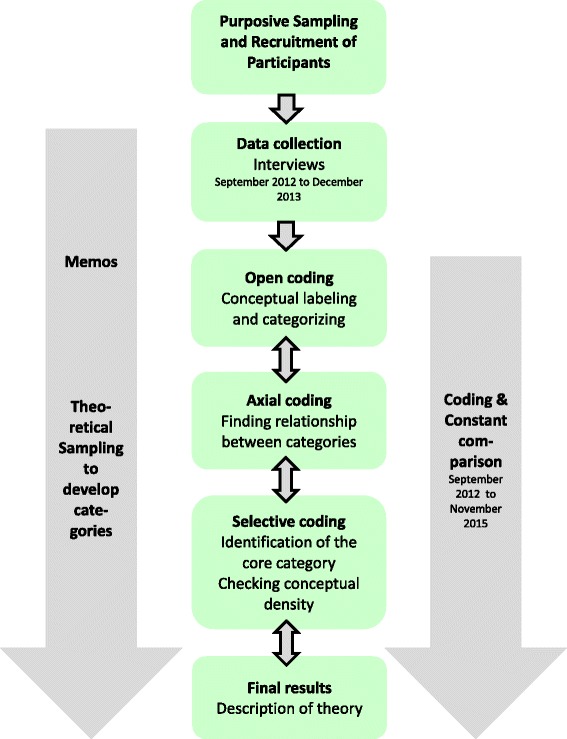
Flow chart of data analysis

Similarities and differences of phenomena and concepts within the interviews were assessed in an iterative comparative process (Fig. 1). To extract the various perspectives on the meaning of the data, we analysed the interviews in a team of four researchers with different professional backgrounds (nursing science, occupational therapy, gerontology, medicine). In regular meetings of the entire research group we discussed the findings and presented them additionally to staff external to the core analysis team. Open coding identified GPs’ expectations, experiences and strategies. Axial coding was used to cluster and organize the data. We used the coding paradigm as a heuristic tool developed by Corbin and Strauss [22]. As the recruitment based on method of theoretical sampling was not feasible we followed the principles of theoretical sampling and constant comparison method within our analysis until our categories were saturated. Once the core category had been identified by selective coding we additionally reviewed the literature relevant to the emerging theory.

## Results

### Core category “productive performance” of home visits and its context

“Productive performance” was identified as the core category of GPs’ perception of their nursing home visits. GPs aimed to achieve “productive performance” by balancing individual effort and reward. The feeling that the visit was worthwhile served to strengthen this balance. This process was influenced by their evaluation of the benefit for their patients, their personal commitment, the maintenance of their own medical practice as an independent business and the wish to provide care in nursing homes, with an overall economic thought kept in mind.

*“so that a reasonable mixed calculation is somehow profitable in the end. You can´t care for too many residents in nursing homes who, ah, disrupt the schedule so much so that surgery appointments aren’t possible” (AA6/42)*

### Types of home visits

The “productive performance” approach was found in the context of four different types of home visits: 1. on demand, 2. periodic, 3. nursing home round and 4. based on ad-hoc-decisions. These different types of home visits could be differentiated by their regularity, plannability, routine in time-scheduling as well as content-related planning and practical preparation. An overview of the types of home visits and the paradigm model of “productive performance” is given in Table 3.

**Table 3:** Overview of “productive performance” in types of home visits, influencing conditions and causes, strategies and consequences

Types of home visits	Influencing conditions and causes	Strategies to achieve “productive performance”	Consequences of “productive performance”
• Visits on demand • Periodic visits • Nursing home round • Visits based on ad-hoc-decisions	• Nurse characteristics • Resident characteristics • Nursing home environment	Preparing strategies • Scheduling and planning • Preparing On-site strategies • Gathering information • Seeking nurses´ attendance • Decision making • Taking care of the resident Investing strategies • Instructing and teaching • Dealing with documentation • Providing information	• Satisfaction • Annoyance • Disrespect • Avoiding contact to the nurses • Give up care

**Visits on demand** were the least plannable, but required by a change in resident health status as perceived by the residents themselves, their relatives or nurses. Residents or their relatives informed the GPs or reported the change to a nurse, who decided in their filtering function to contact the GP practice by phone or fax, depending on the perceived urgency and existing arrangements with the GP. Usually the practice assistants received this information from the nursing home and forwarded it to the GPs.

*“the nursing home calls me, the patient complains of foot pain, I would like to check that”(BA2/7)*

Depending on the health problem and previous experience with the nursing staff, the GP then decides whether to visit the nursing home or to call for an ambulance. This initial referral to hospital instead of a home visit was also a part of “productive performance” because the conditions in the nursing home are not appropriate for severe, acute health problems or specific diagnostics.

*“the resident has dyspnea, ah, I won´t visit but will call the ambulance instead. There´s no reason to attend because I would be on my own without the necessary equipment or staff to assist me“(CA3/4)*

Although **periodic visits** were generally more routine for the GP, they were not automatically perceived as more productive. Periodic home visits are planned and focus on regularly monitoring the health of residents with chronic diseases. They are characterized by short duration and unidirectional communication: the GPs inform the nurses only briefly of the reason for the visit. Such advance scheduling led to “productive performance” as some activities could also be delegated to the practice assistant, e.g. blood sampling.

*“I personally do the rounds in two nursing homes, today I saw about 40 residents on two wards in one nursing home between 7:30 and 11:30. I went there, was expected, the nursing staff were waiting for me and had prepared a book with the questions of the day. I advised them to set up this book” (AA1/3)*

Caring for at least two residents in one nursing home is often associated with a **nursing home round**. This is perceived to be similar to a hospital ward round in which the GP visits all his residents, one after another. GPs adapted this process to the residents` needs; they asked for the health update and monitored existing chronic diseases. This type of visit followed a highly structured, time-saving routine.

*“first I meet up with the nurse (…) we sit down together and talk theoretically who should be visited and what I have to sign and so on. After that I go through the nursing home to the individual residents” (AA10/54)*

The **visits based on ad-hoc-decision** represented a hybrid of the demand and periodic visit and depended on the particular situation. The decision to extend the visit was influenced by the other residents´ health status, the perceived urgency and operational requests such as prescriptions and signatures – which was renegotiated in every visit.

*“depends whether there was something out of the ordinary”(CA1/2).*

### Influencing conditions and causes

Nursing home residents and nursing staff represented the most important counterparts during the home visit. Additionally structures within the nursing home and general conditions of the health care system influenced the “productive performance”.

### Nurse characteristics

Apart from the individual characters, nurses’ qualifications influenced “productive performance”. Wide variations were perceived by the GPs in the level of commitment, formal qualification and the nurses` need for reassurance by the GP. GPs believed these aspects to be influenced by requirements of the German health care system e.g. quality controls of Review Board of the Health Insurance Funds. GPs preferred nurses experienced in hospital care – which is very unusual for geriatric nurses - as well as nurses performing management functions (charge nurse).

*“for example I currently undertake wound management for a resident who has very bad …problematic wounds and I do this exclusively with the head nurse” (BA1/4)*

The more a nurse fulfilled GP expectations, the better GPs experienced the collaboration and the “productive performance” during the visit. GPs did not like being ignored by the nursing staff or having to wait if nurses continued their own tasks or were unfamiliar with a situation. Moreover the “productive performance” was decreased by temporary staff and/or insufficient German language skills. Nurses’ qualifications also influenced the time required for the visit.

*“well, [the claiming of time] varies between the nursing homes, depending on nurse or the geriatric nurses qualifications“ (BA3/32)*

Nurses influenced GPs` “productive performance” by acting as a source of information. Many nurses knew the residents well, including their actual physical health status i.e. blood sugar levels, and/or mental and social situations. In this context, a clear role assignment by the nursing home eased the contact. Furthermore nurses represented a familiar face to residents who are new in a GP´s care, or suffering from cognitive deficits, or were otherwise unable to talk to the GP about their symptoms and requests.

*"How are the blood glucose levels? (…) This often gives the opportunity to obtain a lot of information from the [nursing staff] (…) often very precise and exact statements about the mental status of the patient, especially from the experienced nurses. How well [the resident] is integrated, are there any changes or mental problems? This is also often very helpful” (CA1/10)*

Furthermore nurses represented a familiar face to residents who were new in a GP´s care, suffered from cognitive impairment, or were otherwise unable to talk to the GP about their symptoms and requests.

*"one of them suffers from Alzheimer´s disease, the other is over100 and also has dementia. I only visit them with the same nurse because [the resident] is anxious with people she doesn´t see regularly” (BA4/54)*

Contact to the nurses is experienced within a framework of the visit to the resident. GPs viewed the nurses as navigators and partners. The ideal nurse was friendly, well prepared on time, knew the current health status of the residents and had time to accompany the home visit (or even the chart round) immediately with her full attention.

*“where someone is quasi waiting for me” (AA3/14)*

But often the reality is different. GPs had to seek the nurses in their office, in the residents´ rooms or by using the nurse call system with variable success, which lessened their “productive performance”.

*“and then you initially have to search for a quarter of an hour until you find a nurse” (CA5/46-48 )*

### Resident characteristics

GPs experience ambivalent emotions towards the nursing home and its residents such as helplessness or not being able to please everybody. GPs sometimes encountered aggressive challenging behavior of the residents during their visits. The residents were viewed as “occupants” (BA1/127) who sat around “one is babbling, the one next to her is screaming and the rest of them sit around, staring” (BA1/131) consequently GPs were glad to leave the “musty atmosphere of severe illness” (AA3/33). In contrast, GPs could also acknowledge that the residents were very pleased about their visit. GPs enjoyed this special role providing a change in routine and as a person to be respected and highly regarded. This mix of appreciation and burden reflects the GP ambivalence.

*“people are glad to be visited so they don´t have to go to the practice. Many of them couldn´t even come. Well, this is always really pleasant” (AA6/22)*

### Nursing home environment

GPs continually had to adjust to the different structures and organization of the nursing homes they visited. Standards of visiting did not seem to be known by the nursing staff and varied from one home to another, affecting “productive performance”. The nurses´ shift system increased the number of contact persons and therefore the risk of communication barriers.

Working in a nursing home was compared with treatment and collaboration with nurses in the hospital, which had a positive connotation for the GPs.

*“well, the opportunity to work with a team of nursing staff. It’s a bit like that in a hospital. In the practice, you are on your own, I am alone with my patient and [in the nursing home] the nurse is attending and this seems to me like a hospital atmosphere – I like that”(AA10/100)*

### Strategies to achieve “productive performance”

**Fig. 2:**
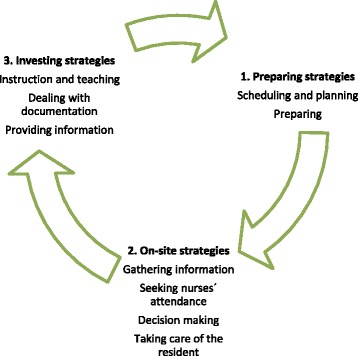
Strategies to achieve "productive performance"

GPs applied different strategies to achieve “productive performance”. These could be classified into three types: preparing strategies, on-site strategies and investing strategies (Fig. 2). Preparing strategies were pre-visit preparations to facilitate “productive performance” prior to entering the nursing home. On-site-strategies included all procedures during the visit and were influenced by the visit partners, namely, the nurses and residents. At the end of the visit, GPs often undertook investing strategies (providing resources and/or information) attempting to ensure fewer requests prior to the next visit. All strategies served to strengthen GP feelings that visiting nursing homes were a worthwhile “productive performance”. While not all the strategies necessarily occurred at each home visit, each individual element reflects the richness of the data.

## Preparing strategies

### Scheduling and planning

Time-scheduling and content-related planning in advance of the actual nursing home visit contributed to the “productive performance” and represented the start of every nursing home visit. GPs determined the time, length and structure of the home visits in a way they considered most productive. They also made appointments with nurses and expected strict adherence to the time schedule. Some GPs agreed quid pro quo to adjust their time to the nurses´ daily routine if they rated the meeting important. Flexibility with regard to standard or regular visiting routines often provided a better chance to meet a nurse with enough time for them and was experienced as more relaxed.

*“the typical days for home visits in City1 are the afternoons of Wednesday and Friday. You can imagine, on these two days, one or two nurses and ten GPs are ready to visit. I think it´s more relaxed to visit the nursing home in the morning when no one else is there. I have all the time in the world, there is no pressure (laughing) the nurses normally have finished breakfast and that has proven its worth“(CA5/20)*

### Preparing

For a “productive performance”, GPs expected the nurses to prepare for (in particular) periodic home visits and nursing home rounds, namely to collect requests and send a fax or an email in advance.

*“we ask the nurses for a fax detailing which of the residents require a visit so that we know who requires a smear test or if suture material is needed, or anything else that we additionally have to bring . The list is sent back a day before the visit, mostly in time. And I take all the relevant patient-records with me” (AA2/8)*

Nurses´ frequently failed to send the preparation list, which generally led to a reserved and bleak atmosphere during the home visit, clearly diminishing the “productive performance”. GPs expected to receive all important information in advance without needing to prompt. For example if GPs were not informed about a resident´s hospital admission, they would arrive unnecessarily to the home visit.

*“I immediately expressed my upset that I want to know something like this [hospital admission]” (CA4/34)*

The practical preparations of GPs for a “productive performance” included taking everything that might be needed, e.g. blank forms and prescriptions. Individual scheme for preparing the home visit helped the GP to remain informed. This included GPs periodical chart review of residents medication, need for signatures or referrals to other health professionals.

## On-site strategies

### Gathering information

An important strategy of achieving “productive performance” during the visit was gathering information about the residents. Arriving at the nursing home, the GPs initially would seek to get an overview. GPs initially went to the nursing office either to catch up directly a nurse or to check the medical records. For periodic home visits and nursing home rounds in particular, the chart-round (in which the residents´ medical issues are discussed based on their charts) contributes information and establishes appropriate care requirements and confirms or disproves any spontaneous decisions *“depending on particular requests”(CA1/2).* GPs considered it important to receive an impression of the resident and verification of the information they had received in advance. Perception of residents´ health status could differ between GPs and nurses. GPs expected nurses to share their perceptions of residents´ health. *“What is the matter?”(AA8/2)* was one of the leading questions used so that an acute problem, a medical question or a symptom could be rated during a structured preparation ahead of meeting the residents.

### Seeking nurses´ attendance

Another strategy was to choose specific contact persons to obtain the necessary information and achieve continuity or assistance depending on the health and cognitive status of the residents. GPs generally insisted that this specific nurse the home visit and therefore made appointments in advance. Moreover, nurses’ attendance from the beginning of the visit avoided delays when there was a sudden need for assistance or information. Nurses in attendance had the opportunity to quickly perform tasks resulting from GPs´ decisions.

In some cases, depending on the doctor-resident-relationship and the GP role as a trustworthy, familiar person, a nurse’s attendance however could be perceived as unhelpful to a “productive performance” and their attendance not desirable. A confidential atmosphere in a private conversation boosted the GPs´ relationship with the resident.

*“they [the residents] tell me things they won´t tell to other people and therefore nobody else should come along” (AA7/118)*

### Decision making

We found several dimensions to the decision making strategy, ranging from a basic treatment course to complex decisions for holistic problems. To be able to make decisions, the reported situation was matched with the medical history and laboratory results. Particularly in palliative situations or before decisions of admission to the hospital, GPs communicated with nurses, residents and relatives, before arriving at a final decision. In conflict situations, for example, they would give instructions that would affirm their authority.

*“so I decided with the others and against the explicit instructions of [the head nurse] , that [the resident] will stay here. I cannot do otherwise” (CA5/128)*

### Taking care of the resident

For GPs, taking care implied medical care as well as being a social contact for the residents. Many GPs regarded listening to residents part of their medical remit. Even on requested visits, GPs would often take time to talk about non-medical topics with the resident e.g. which family member came to visit or how to spend Christmas – this was not perceived as contrary to a “productive performance”.

*“Finally I talked to [the resident] for only twenty minutes and otherwise we didn´t change anything concerning the therapy? And then she was also satisfied” (AA/50-54)*

GPs viewed their role as not strictly limited to medical care but also includes social contact and caregiving.

*“however they are socially isolated to some degree. Relatives don´t come around and sometimes there aren’t any friends any more. In this respect, I am just a conversation partner and simply listen to one or other minor problem which is sometimes outside the medical sphere”(CA2/28)*

## Investing strategies

### Instructing and teaching

“Productive performance” could be strengthened through teaching and instruction of nurses to achieve a common appreciation of residents’ health status as well as facilitating nurses’ autonomous decisions. While with the resident, GPs would give advice and instruct the nurses as well as offer further training to improve their knowledge and to enable them to assess the health status of the resident in an appropriate way. This was considered useful particularly when nurses either had poor medical competencies or felt pressured by organizational constraints, e.g. quality management. Such teaching is consequently expected to strengthen the nurses’ knowledge and to result in fewer requests of unnecessary home visits.

*“I observed while she [the resident] was being tested and recognized that she wasn’t inhaling correctly (…) I took the opportunity to go through all details about the application with the nurses” (AA9/68-76)*

### Dealing with documentation

GPs experienced pressure when dealing with documentation. Nurses insisted on written prescriptions referring to the quality control regulations of the German Health Insurance Medical Service. In the opinion of the GPs, these do not need to be followed to the letter. GPs were found to adopt one of two possible strategies to deal with documentation demands. On the one hand such details were considered *“annoying and time-consuming” (CA3/26), “produces work for nothing” (AA6/108), “recording is rewarding” (AA8/66), “like a writing-jackass” (AA8/66).* Such GPs felt their ”productive performance” was affected by the paperwork they considered to be senseless. On the other hand, GPs decided that complying in order to do a favour to the nurses was a good investment for “productive performance” and further collaboration. Documentation was sometimes also seen as a supporting work tool, increasing transparency and clarifying prescriptions.

*“they [nurses] print it for me to sign so that it is documented and then taken forward” (CA/25)*

The heterogeneity of record systems, pen-and-paper or electronic versions, complicated the situation. GPs had no access to every database or keys to filing cabinets and therefore needed a nurse as an intermediary.

### Providing information

GPs would leave prescriptions and information to ensure continuous care between visits and this therefore indirectly strengthened “productive performance”.

*“well, I try to do it in a personally way, that seems to be the best for me. If that doesn´t work because they [the nurses] are in the resident rooms, I go to their office and put it properly on the table”(AA2/87)*

Clear orders and instructions avoided inquiries between visits which would reduce the “productive performance”. Contact however was considered unnecessary if there were no new prescriptions or changes in therapy and GPs then generally left the nursing home without speaking to the nurses.

## Consequences of “productive performance”

### Satisfaction

Thorough preparation of home visits (preparing strategies) and a good, valued interaction with the nurses on site was reminiscent of the “the hospital atmosphere” (AA10/100). Direct bidirectional communication led to a positive team environment that differed to the daily routine of the medical practice *“That´s fun! (…) It’s another type of working, eh?” (AA6/150). If the on-site strategies worked, nurses´ attendance created a communicative and supportive atmosphere for all stakeholders.*

*“it represents a sensible ward round, that both professionals have the same level of knowledge. This happens best when the same is seen at the same time” (AA5/21)*

The satisfaction which results from this atmosphere might even lead to familiar conversations beyond work tasks.

*"and I even get a coffee and some cookies and we [the nurse and I] talked privately, something normally that there is no time for. I found that was very nice. (…) and we chatted a bit."(AA2 / 51–53)*

The GPs own satisfaction with the work was perceived as corresponding with the satisfaction of the residents. The strategy of taking care of the resident led to the feeling of having done something meaningful for them. When the strategies performed by GPs to achieve a “productive performance” are successful they were expected to lead to fewer inquiries for on demand home visits.

*“finally I talked to her [the resident] for twenty minutes and otherwise we didn´t change anything, eh? Therefore she was satisfied” (AA/50-54)*

### Annoyance

If the strategies for a “productive performance” did not work, annoyance of the GP could be the first reaction. If they perceived nurses as unorganized or lacking structure, GPs felt their visit to be superfluous and a waste of time. They expected nurses to share their perception of the resident health status. From GPs view, nurses should create communication strategies within the nursing staff team to avoid unnecessary GPs´ visits.

*“the resident had a swollen hand and I arrived but I couldn´t see any swelling. I was upset, I can tell you (…) And none of the nurses could even say why they called me” (AA7/94-96)*

### Disrespect

Unstructured preparation and information as well as a lower qualification increased GP disrespect for the nurses. One manifestation of this was that GPs did not regard all of the nurses to be capable of understanding medical issues.

*“and in the time required for me to spell Hydrochlorothiazide for them [the nurses],it would have been faster if I had written it down myself” (AA1/129-133)*

### Avoiding contact to the nurses

When the visit resulted in *“no new prescriptions or changes in therapy”(CA3/18)*, some GPs avoided further contact to the nurses, as they deemed it unnecessary and left the nursing home. Searching and/or waiting for nursing staff or confronting them with a potentially unwanted decision affected “productive performance”. In situations perceived as poor collaboration, GPs sometimes chose to avoid contact to the nurses altogether or consulted the nursing home management.

*“on one ward I experienced very poor collaboration, so I decided not to go to the nurses’ office any more. I just collected the documents I needed, went to the residents and communicated by fax (…) that is definitely not good behavior for a physician“ (AA8/128)*

### Give up care

In cases where the conflicts could not be solved, GPs decided to give up providing care at that nursing home.

*“the care became more and more disputed, so I asked to be replaced by another GP” (AA07/213)*

## Discussion

### Principal findings

This in depth study in the context of nursing home visits revealed that “productive performance”, a balance of individual effort and reward, was the key issue for GPs visiting nursing homes. We identified four types of visits and were able to show that nurse and resident characteristics, as well as the nursing home environment, were influencing factors. GPs clearly sought to actively influence the structure of the visits: They applied strategies to achieve a “productive performance”: starting the visit long before they entered the nursing home and also maintaining indirectly the “productive performance” after leaving its premises.

### Strengths and weaknesses of the study

The strength of our study is the detailed exploration of the nursing home visit using the grounded theory approach [22]. We found rich contrasts of opinions and behaviors of GPs, although social desirability and appealing presentation of GPs (inter-) action during the visit cannot be fully excluded. A larger group of interviewers with diverse professional backgrounds may have been a source of heterogeneity, but at the same time this also enriched the data collection with different precognitions and experiences influencing their questions and understanding. An interdisciplinary team of three researchers coded the data to ensure credibility [21]. The limitations included time or organizational constraints of the parallel interview analyses and the conduction of new interviews for sampling according to the principles of theoretical sampling [22]. Consequently we mainly performed a purposive sampling. However data could be analyzed with regard to high and low contrast in the characteristics of the participants and content of previous interviews resulting in more purposive recruitment.

### Findings compared to other studies and literature

While previous studies have considered GP nursing home visits, these have been ancillary to alternative primary research questions and study designs. Therefore our findings supplement and deepen the knowledge obtained to date.

We found four different types of home visits to nursing homes (visits on demand, periodic visits , nursing home round and visits based on ad hoc decisions) Theile [20] thus distinguished between supportive and urgent and routine home visits. In Theile’s findings, the routine visits supported GPs monitoring of the resident and were perceived as the least challenging by them. According to our findings, visits on a routine basis (periodic visits and nursing home rounds) had high productiveness in contrast to more urgent visits (visits on demand and ad hoc decision-based visits) which were experienced with a high workload that negatively affected the “productive performance”. Considering these findings and our results, “productive performance” is determined by the perceived workload in addition to regularity, plannability and routine. Also Block et al. [24] have found that GPs generally sought to ease their workload, but were unable to indicate the strategies whereby this was achieved. Our core category, “productive performance”, was influenced by several conditions and causes. Nurse characteristics were crucial for “productive performance”. Similar to other studies, GPs had a positive experience working in cooperation with a friendly, familiar, reliable and dedicated nurse with professional competence [24, 25], and expected nurses to meet this ideal in order to achieve a “productive performance”. “Productive performance” was also influenced by residents´ characteristics. When residents were pleased about their GP visit, GPs were also more satisfied with the situation and their role; when resident behavior was challenging, GPs felt burdened. Other studies have described emotional GP perspectives towards the nursing home either as places of resignation, incapacitation, loneliness and despair [20] or as an enjoyable, important and meaningful work for the GP´s [26], but not the ambivalence we found. The resolution of such ambivalent emotions may influence “productive performance” and should be explored in further studies. Different nursing home environments contribute to a feeling of imbalanced efforts and rewards, as GPs must adapt to the respective structures of each nursing home. GPs do not have much influence on organizational issues, staffing or corporate policy and therefore felt less effective in managing their “productive performance”. Other authors found GPs struggled with nursing staff shortages (19). GPs used several strategies to achieve “productive performance”. Similar to our findings other authors also found that GPs established strategies before the visit [25]. Seeking nurse attendance was one on-site strategy of our interviewees; GPs also chose specific contact persons and made appointments with them – small, but efficient interventions, depending on the individual effort of a GP. Theile [20] as well as Struppek [25] also cited nurse attendance as an essential component of the visit for information exchange, interaction and assistance. GPs experienced nurses as supportive during the visit, but appreciated that they themselves were disruptive for the nursing routines [25]. Block et al. [24] found nurses rarely accompanying GP visits due to different time schedules, but the lack of time was cited as an obstacle for collaborative work. GPs and nurses seemed to have dealt with the reported deficiencies [24], which is indicative could also be regarded as a type of strategy. Another important on-site strategy for our GPs was to gather information. According to our data, chart-reviews added information, but the GP´s own impression of the resident had more emphasis. In another study, chart reviewing was also considered a time-saving opportunity instead of visiting every resident personally [20]. We also detected many emotive and mainly negative statements about “dealing with documentation”. While this issue may represent an outlet for GPs´ emotions, GPs also tried to minimize such non-conforming frustrations toward the residents and nursing homes in general. Achieving “productive performance” had positive consequences for the GPs as they were more satisfied with different aspects of their work. In a qualitative study of GP perspectives on nursing home care in Sweden, GPs also considered their work in nursing homes as “enjoyable, important and meaningful” [27, 28]. Consistent with this, a number of consequences became apparent when “productive performance” could not be realized: annoyance, disrespect, avoidance of contact to nurses and give up care shown in our theory and these were comparable to GP passivity in conflict regulation found by Block [24]. Open conflicts could threaten collaboration with the nurses and therefore led to avoidance passivity. GPs then preferred to give up care at a home rather than to work out a solution for all parties. In our data, nurses appear as a key person in the majority of the strategies to attain “productive performance” next to minor e.g. supportive and service roles. GPs see nurses in contradictory roles as preparer, informer, partner and student. In a Swedish study GPs gave the nurses the role of the mediator, negotiator and/or coordinator [26]. Different roles of GPs in nursing homes were described in other studies: supervisor [20], consultant or conductor [27]. Roles extracted from our data might include the decision-maker, the teacher and the carer, which could mostly be related to Modin´s conducting roles [27]. In our study GPs described themselves as preparer, informer and partner within their strategies. Nurses` seemed to have a more positive attitude towards collaboration than GPs [28]. GPs regarded themselves as highly independent [27, 29, 30]. Cooperation with the nursing staff during the nursing home visit has also been broadly discussed in other studies [20, 25, 26]. An interesting additional observation from our interviews was that GPs drew a parallel between their work in nursing homes and the hospital setting, which has also been described in other studies [20, 26]. This comparison was positive for the GPs not at least with regard to “productive performance”. Ward rounds in hospital settings and the nurse-physician-relationship in this context have been well studied [31–33], however differences and parallels between the settings have to be considered carefully.

### Implications for research and practice

“Productive performance” is the main theme of German GPs with regard to their nursing home visits. Our results suggest that it would be beneficial if nursing homes could create and provide structural arrangements which enable the GP and nursing staff to perform more productively. Examples could include nursing homes providing defined procedures both for planning and preparing GPs’ nursing home visit and also during the nursing home visit itself by ensuring provision of an accompanying nurse. If GPs do not consider their performance sufficiently productive, they could also be motivated themselves to structure their visits better, to prepare it differently or to offer training to nurses. Truscott proposed the negotiation of a written, standardized contract of responsibilities and communication patterns for collaboration in nursing homes [6]. A recent German law, adopted in 2012, seeks general agreement for interprofessional collaboration on a structural basis, but has to date only been implemented in a few model-projects. Moreover these coordinating activities need to be included in the contract negotiations with the health insurance fund alongside the GP payment system [34].

Visits to the nursing home should be defined independently in structural and organizational aspects as compared to other types of home visits. In the Netherlands and France, specialized GPs are in charge of care of nursing homes or co-ordinate and perform an advisory role between the nursing homes and the residents´ regular GPs [35].

Our findings could also serve as the basis for the development of educational concepts on “the nursing home visit” as part of the vocational training for GPs and nurse education and training. This may result in a deeper understanding of the GP perspective. This could underpin closer collaboration, constructive discussions and better integrate the GP into the team of nursing home staff and possibly. Moreover it will be helpful to anticipate the scope of GPs’ influence for young professionals also additional professional groups e.g. social support or therapists. Indeed, the evaluation of the views of these other stakeholders involved in nursing home visits, namely, residents, relatives and nurses are in preparation in the interprof study. These will complete the detailed in depth view on the nursing home visit. Such perspectives and wishes of all the involved groups will ultimately facilitate better collaboration in nursing homes and improve resident care. Finally further research is needed to explore the impact of “productive performance” on GP job satisfaction as described in [35] and on resident-related outcomes.

## Conclusion

Our theory and findings of GP views on their nursing home visits will be useful for future research by various health professional researchers and nursing home management to generate a deeper understanding of GP perspectives. Collaborative working and open discussions between all care providers should facilitate visit preparation and on-site procedures as well as training, dealing with documentation and the provision of information. Such a scenario would be anticipated to improve interprofessional collaboration and ensure a high quality of care.

## Abbreviations

GP: general practitioner
